# Circular RNAs associated with a mouse model of concanavalin A‐induced autoimmune hepatitis: preliminary screening and comprehensive functional analysis

**DOI:** 10.1002/2211-5463.12981

**Published:** 2020-10-14

**Authors:** Yang Liu, Zhencheng Li, Jianheng Hao, Hao Chen, Tiezheng Hou, Huiqin Hao

**Affiliations:** ^1^ College of Basic Medical Sciences Shanxi University of Chinese Medicine Jinzhong China; ^2^ Basic Laboratory of Integrated Traditional Chinese and Western Medicine Shanxi University of Chinese Medicine Jinzhong China

**Keywords:** autoimmune hepatitis, circRNA, concanavalin A, gene ontology, KEGG, microarray

## Abstract

Without treatment, autoimmune hepatitis (AIH) often leads to cirrhosis, liver failure and, in some cases, death. However, the pathogenesis of AIH remains incompletely understood. Here, we explored the relationship between differentially expressed circular RNAs (DECs) and development of AIH by obtaining an expression profile of DECs in a concanavalin A‐induced AIH mouse model by microarray. In total, we identified 27 DECs; the host genes of these DECs were annotated with 140 Gene Ontology terms and 19 pathways, revealing potential roles in the metabolism of cellular ions and regulation of protein expression, as well as possible involvement in endocytosis and apoptosis. We constructed a circular RNA–microRNA network that was used to infer that a *mmu_circ_0001520/mmu‐miR‐193b‐3p/MAPK10* network may be associated with the occurrence of AIH. These findings may help lay the foundation for validation of the potential roles of circular RNAs in AIH.

## Graphical abstract

Here, we investigated differentially expressed circular RNAs in a concanavalin A ‐induced AIH mouse model with microarray, identifying differentially expressed circular RNAs that may play crucial roles in the pathogenesis of AIH. These candidates may have potential for development into novel therapeutic biomarkers for this disorder.

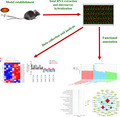

**Abbreviations**
AIH: autoimmune hepatitis
BP: biological processes
CC: cellular components
ceRNA: competing endogenous RNA
circRNA: circular RNA
Con A: concanavalin A
DEC: differentially expressed circular RNA
FC: fold change
FDR: false discovery rate
GO: Gene Ontology
IL: interleukin
KEGG: Kyoto Encyclopedia of Genes and Genomes
MAPK: mitogen‐activated protein kinase
MF: molecular functions
miRNA: microRNA
mTOR: mammalian target of rapamycin
PCA: principal component analysis
qRT‐PCR: quantitative real‐time polymerase chain reaction
TCR: T cell receptor
Th: helper T cell

Autoimmune hepatitis (AIH) is a type of classical autoimmune disease in which the liver is the main target organ [1]. This immune‐driven self‐reactive disease occurs in any ethnicity and at any age worldwide, with a female preponderance [2, 3]. It is characterized serologically by hyperglobulinemia and presence of serum autoantibodies, as well as histologically by lymphocytic infiltration and interface hepatitis in liver [4, 5]. The etiology of this immunological disorder is likely to be related with the genetic and environmental factors (including viruses, bacteria, alcohol) [6, 7]. Most AIH cases manifested as chronic processes with no specific clinical manifestations, although a small number of patients presented with acute hepatitis [8, 9]. Without treatment, AIH often leads to cirrhosis, liver failure, or even death [10]. However, up to now, the exact pathogenesis of AIH has not yet been illustrated [11].

Circular RNA (circRNA), which has been identified as a newfound class of noncoding RNAs, is widely present in different cells [12]. Different from linear RNA, circRNA is a closed loop formed by covalent connection of 3′ end and 5′ end, and without 5′ to 3′ polarity and polyadenylation end [13, 14]. It is due to the closed structure that the circRNAs are resistant to degradation of RNA enzymes and are well expressed in the cytoplasm steadily[15]. One of the most essential biological activities of circRNAs is acting as a ‘microRNA (miRNA) sponge’ or ‘competing endogenous RNAs’ (ceRNAs) to regulate the expression of encoding genes indirectly [16, 17]. Accumulating studies on circRNAs have made major progress in elucidating the pathogenesis of different diseases [18, 19], also including autoimmune disease, such as systemic lupus erythematosus and rheumatoid arthritis [20, 21, 22]. However, the relationship between differentially expressed circRNAs (DECs) and the development of AIH has not been reported.

Hence the DECs in an AIH mouse model induced by concanavalin A (Con A), a well‐established model for closely mimicking the process of AIH in human [23], were screened using microarray chip for the first time. The functional assignment of these DECs was systematically analyzed with bioinformatic methods herein, to offer state‐of‐the‐art therapeutic strategy for this worldwide hepatitis.

## Materials and methods

### Ethics statement

Adult male C57BL/6 mice (25–28 g) were obtained from Vital River Laboratory Animal Technology Co., Ltd. (Beijing, China). The rules of National Institutes of Health *Guide for the Care and Use of Laboratory Animals* (NIH Publications No. 8023, revised 1978) were complied with in this study, and the animal experiments were approved by the Ethics Committee of Shanxi University of Chinese Medicine (Permit Number: 2019LL41). The mice were housed under controlled temperature (21–24 °C) and humidity (40–60%), 12 h dark/light cycle, and feeding *ad libitum*.

### Reagents and chemicals

Con A (batch number: C8110) was gained from Solarbio Science & Technology Co., Ltd. (Beijing, China). Chloral hydrate (batch number: A600288) and UNlQ‐10 Column Total RNA Isolation Kit (batch number: B511321) were obtained from Sangon Biotech Co., Ltd. (Shanghai, China). Maxima Reverse Transcriptase (batch number: EP0743) was bought from Thermo Fisher Scientific (China) Co., Ltd. (Shanghai, China).

### Animal experiment

AIH mouse models (*n* = 4) were established by intravenous injection of Con A solution (15 mg·kg^−1^, dissolved in pyrogen‐free saline), and another four mice taken as the control group were injected with pyrogen‐free saline via the tail vein. All mice were sacrificed with an intraperitoneal administration of pentobarbital sodium solution at 8 h after the injection, and their livers were collected under low temperature and sterile conditions. These hepatic tissue samples were homogenized immediately for extracting total RNA with TRIzol solution, and the integrity of the extracted RNA was assessed with Agilent Bioanalyzer 2100 (Agilent Technologies, Boston, MA, USA).

### Microarray hybridization and data analyses

circRNA microarray analysis was conducted by OE Biotechnology Co., Ltd., (Shanghai, China) with the OE biotech Mouse Microarray 2018 (Agilent‐085631, containing probes for 14 747 circRNAs from the ‘circBase’ database) in this research. The sample labeling, microarray hybridization and washing were performed based on the manufacturer's standard protocols. The Agilent Scanner G2505C (Agilent Technologies), feature extraction software (version 10.7.1.1; Agilent Technologies) and genespring software (version 14.8; Agilent Technologies) were used to accomplish the scanning and basic analysis with the raw data. To begin, the raw data were normalized with the quantile algorithm, and the standardized data were filtered for subsequent analysis provided the conditions that at least 75% of the samples labeled as ‘detected’ were met. The threshold set for DECs was fold change (FC) ≥ 2.0 and *P* < 0.05. Principal component analysis (PCA) and hierarchical clustering were also conducted to show the distinguishable expression pattern of DECs among different samples.

### Quantitative real‐time polymerase chain reaction validation

To validate the microarray results, we chose six DECs (three up‐ and three down‐regulated) for quantitative real‐time polymerase chain reaction (qRT‐PCR) amplification with Step One PULS real‐time fluorescent quantitative PCR (ABI, Foster, CA, USA). Features of the candidate DECs were presented in Table 1. Their primers were synthesized by Sangon Biotech Co., Ltd., and the sequences were listed in Table 2. Expression of these DECs was normalized to *GAPDH* and calculated with the $2^{‐\Delta \Delta C_{t}}$ method [24].

**Table 1:** Features of the circRNAs selected for qRT‐PCR validation.

circRNA ID	Best transcript	Gene symbol	Annotation	Chromosome	FC	*P* value	Regulation
mmu_circ_0000700	NM_175549	Robo2	INTERNAL	chr16	3.68	2.10E−4	Up
mmu_circ_0001335	NM_001081102	Nsd2	UTR5	chr5	3.41	3.72E−2	Up
mmu_circ_0001843	NM_001114119	Qrich1	INTERNAL	chr9	3.37	6.46E−3	Up
mmu_circ_0001816	None	None	INTERGENIC	chr9	−2.21	3.37E−3	Down
mmu_circ_0000607	NM_001145888	Zfat	INTERNAL	chr15	−2.30	4.69E−2	Down
mmu_circ_0001815	None	None	INTERGENIC	chr9	−2.40	9.42E−4	Down

**Table 2:** The sequences of primers used in qRT‐PCR experiments. F, forward; R, reverse.

Genes	Primers
mmu_circ_0000700	F: 5'‐GAGACGATGACATCAGAAGGGT‐3'
	R: 5'‐GGGAGTTACGTTTGTGTTGCA‐3'
mmu_circ_0001335	F: 5'‐GGTTTCTGCTGACCCACTCC‐3'
	R: 5'‐TCTGGTGTCTGCTTCATCTTCA‐3'
mmu_circ_0001843	F: 5'‐GCACATACCAGAATACGGCTC‐3'
	R: 5'‐GCTGAGACCTGTTGTGGAGACT‐3'
mmu_circ_0001816	F: 5'‐TACAATGGACCTTGAGAGCTTGTT‐3'
	R: 5'‐GGAGAACGTAGGGTAGTCAAGCTT‐3'
mmu_circ_0000607	F: 5'‐AACCTTACCTGGATGATTAGTCTTG‐3'
	R: 5'‐AATCCAAAGGGACACTGAAAAG‐3'
mmu_circ_0001815	F: 5'‐CTCCTACAATGGACCTTGAGAGC‐3'
	R: 5'‐AACGTAGGGTAGTCAAGCTTCCA‐3'
mmu_circ_0001520	F: 5'‐CTTTATCTGACAAGTACAGGTGCC‐3'
	R: 5'‐CTTAACTGCATGACCAGAGGC‐3'
mmu‐miR‐193b‐3p	RT: 5'‐CTCAACTGGTGTCGTGGAGTCGGCAATTCAGTTGAGAGCGGGAC‐3'
	F: 5'‐ACACTCCAGCTGGGAACTGGCCCACAAA‐3'
	R: 5'‐TGGTGTCGTGGAGTCG‐3'
MPAK10	F: 5'‐CCGTATGTGGTGACGCGATA‐3'
	R: 5'‐TGGCGAACCATTTCTCCCAT‐3'
GAPDH	F: 5'‐CTCGCTTCGGCAGCACA‐3'
	R: 5'‐AACGCTTCACGAATTTGCGT‐3'
U6	F: 5'‐CTCGCTTCGGCAGCACA‐3'
	R: 5'‐AACGCTTCACGAATTTGCGT‐3'

### Functional annotation analyses

Enrichment analysis of Gene Ontology (GO) was implemented with the method of hypergeometric distribution algorithm to determine the biological outcomes of the host genes of all DECs [25]. A *P* value <0.05 was set as the cutoff for selecting significantly enriched functional GO terms, including cellular components (CC), molecular functions (MF) and biological processes (BP). Hypergeometric distribution calculation was used for Kyoto Encyclopedia of Genes and Genomes (KEGG) pathway analysis (Release 85.0, January 1, 2018, https://www.genome.jp/kegg/). Each pathway was arranged in ascending order according to *P* value, and a lower *P* value represented higher correlation between the pathway and target genes. The false discovery rate (FDR) was applied to the multiple testing corrections of raw *P* value, and the recommended FDR ≤ 0.05 was the threshold for selecting significantly enriched GO terms and pathways. The GO terms and pathways in which these DECs probably annotated were visually displayed with cytoscape software (version 3.7.2, http://cytoscape.org/; NIGMS, Bethesda, MD, USA).

### circRNA–miRNA analysis

Based on the differently expressed miRNAs screened in the same model [26], the possible target miRNAs that might bind to the circRNA sequence were predicted by ‘miranda’ with the threshold set at ‘Total Score > 170’ and ‘Total Energy < −25’ [27]. The circRNA–miRNA networks were constructed by cytoscape software to illustrate the circRNA–miRNA interactions. Furthermore, one of the most prominent functions of circRNA is as miRNA sponge and to regulate the expression of target mRNAs; the possible ‘circRNA–miRNA–mRNA’ pairs, which participated in the development of AIH, were also predicted by using ‘TargetScanMouse’ (Release 7.2) [28] and ‘circMIR’ software [29], according to both of the ‘miRNA–mRNA networks’ constructed in the same mouse model (data not shown) and literature research. The expression of the genes (normalized to *GAPDH* or *U6*) in the predicted ‘circRNA–miRNA–mRNA’ pairs was validated with qRT‐PCR assay by being calculated with the $2^{‐\Delta \Delta C_{t}}$ method [24].

### Statistical analysis

Independent‐sample *t*‐test was used to identify DECs with spss 25.0 software (SPSS Inc., Chicago, IL, USA). Statistical significance was considered as *P* < 0.05.

## Results

### Identification and validation

**Fig. 1:**
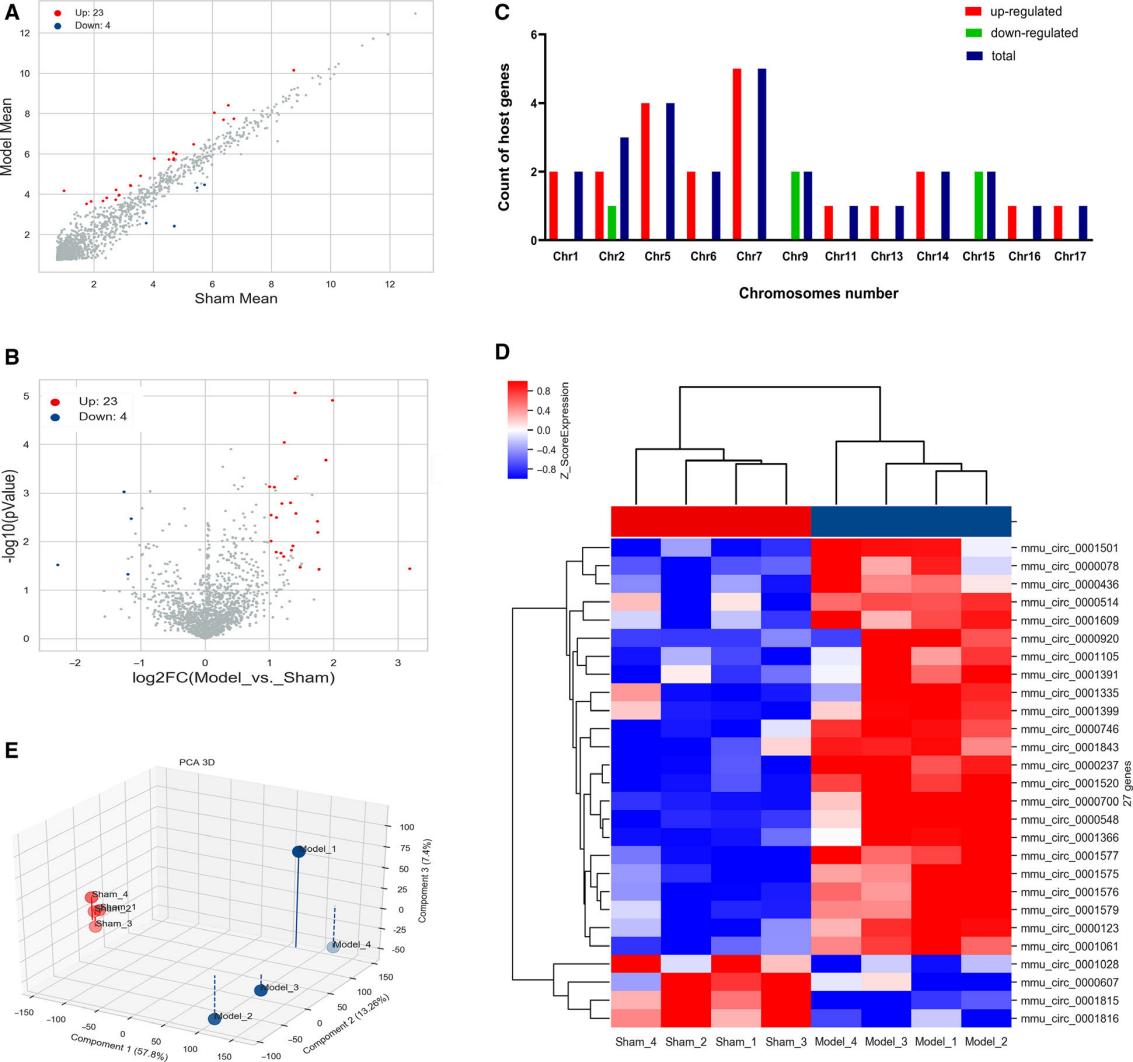
Identification of DECs in a Con A‐induced AIH mouse model. (A) Scatterplot of circRNAs. Red and blue points represented the up‐ and down‐regulated circRNAs between Con A‐induced AIH mouse model and sham group with statistical significance (FC ≥ 2.0 and *P* < 0.05); gray points highlighted the circRNAs expressed but not meeting the threshold. Horizontal and vertical lines signified the average expression level of each circRNA in sham and model groups, respectively. (B) Volcano plots of circRNAs. Red (up‐regulated) and blue points (down‐regulated) in the plot indicated that the FC value and *P* value of the circRNAs were >2.0 and <0.05 between the two compared samples. Horizontal and vertical lines corresponded to log_2_FC and −log_10_ (*P* value), respectively. (C) Chromosomal distributions of DECs. Horizontal and vertical lines represented the count of host genes and chromosome number. Red, green and blue columns separately indicated the numbers of host genes of up‐regulated, down‐regulated and total host genes. (D) Clustered heatmap of DECs. Each column in the heatmap indicated an individual sample (sham 1–4 and model 1–4), and each row represented an individual circRNA. The red and blue shades signified the expression levels of circRNAs above and below the relative expression among all samples. (E) PCA. The red and blue dots signified the individuals in model and sham groups, respectively. The shorter the distance between samples in the same group, the more significant was the separation between samples in different groups, which indicated the more was the experimental reliability and rationality of sample selection.

Compared with the sham group, there were 27 DECs (23 up‐ and 4 down‐regulated) screened out in the model group in accordance with the threshold of FC ≥ 2.0 and *P* < 0.05 (itemized in Table S1). Differential expression of these DECs was visualized with scatterplot and volcano plot in Fig. 1A,B, and the filtered‐out DECs (red points for up‐ and blue points for down‐regulated) were able to be distinguished obviously from the circRNAs under the threshold (gray points). The more the circRNAs were far away from the threshold (red and green lines), the more significant the difference in expression. The most significantly up‐ and down‐regulated DECs were *mmu‐circ‐0000920* (FC = 9.07) and *mmu‐circ‐0001028* (FC = −4.89), respectively. The chromosomal distributions of DECs demonstrated that most circRNAs were transcribed from chromosomes 5 and 7 (Fig. 1C), indicating that abnormity of chromosomes 5 and 7 was closely bound up with the establishment of AIH.

The results of hierarchical clustering analysis disseminated that all mice in the model group were clustered together and differentiated from the sham individuals. The discriminatory power of these DECs was exhibited with a heatmap in Fig. 1D. The red and blue shades represented the up‐ and down‐regulated expression of each circRNA in each subject. According to the results of PCA in Fig. 1E, we also found individuals in the model group flocked together and separated significantly from the sham group. It was indicated that experimental reliability and rationality of sample selection satisfied the conditions for further analysis, and the reproducibility of the data was revealed.

**Fig. 2:**
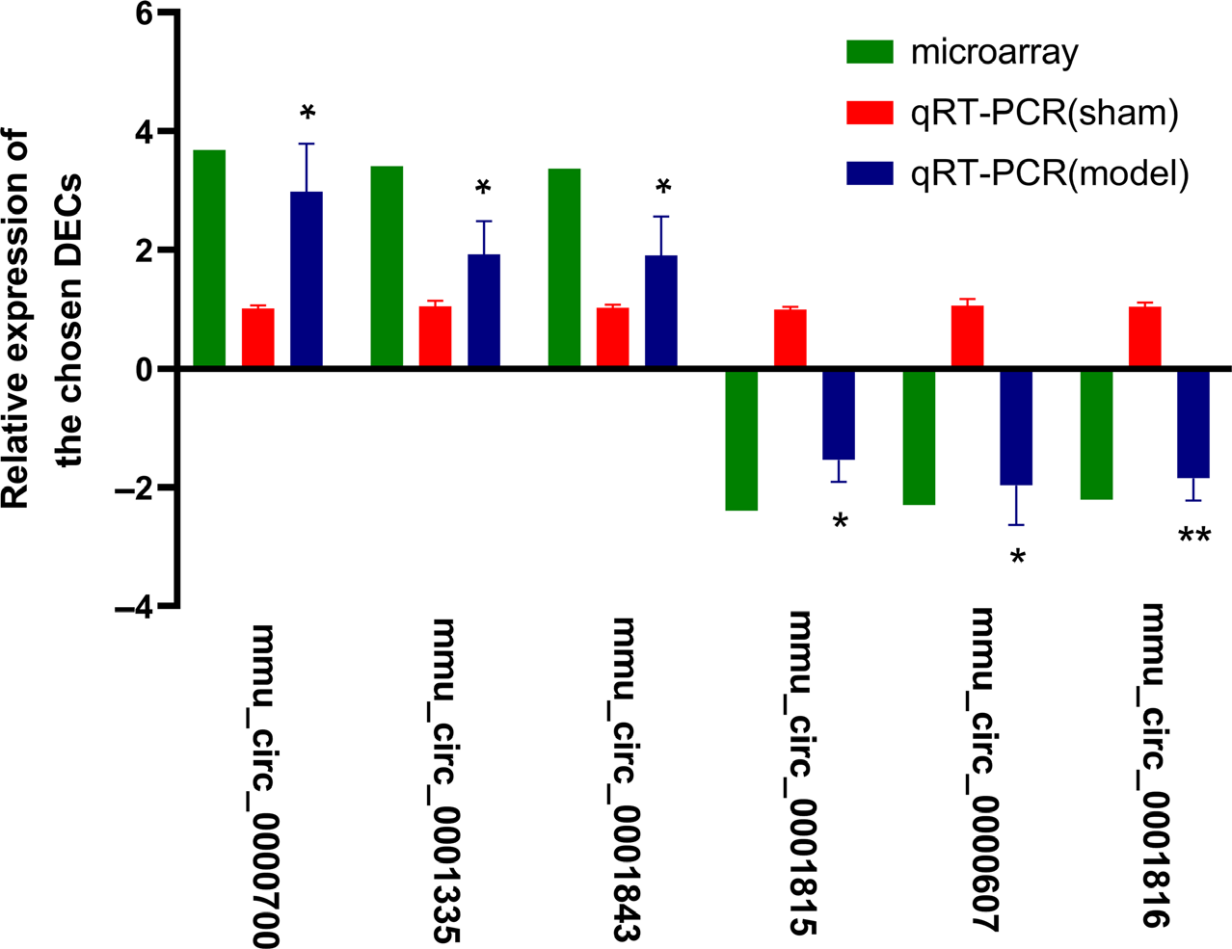
qRT‐PCR validation of the chosen circRNAs. Horizontal and vertical lines represented the chosen circRNAs and their relative expression level (*n* = 4). Red column indicated the results of microarray (FC), whereas green and blue columns reflected the findings in sham and model groups with qRT‐PCR. *GAPDH* was used as an internal control. The data were shown with the mean ± standard deviation (error bars) and statistically analyzed by Student's *t*‐test. *P < 0.05, **P < 0.01, compared with the sham group. *P* < 0.05 was considered statistically significant.

To verify the reliability of microarray results, six DECs (three up‐ and three down‐regulated) were picked out to detect the relative expression with qRT‐PCR assay. Compared with the sham group, the relative expression of *mmu_circ_0000700* (2.98‐fold, *P* < 0.05) in the model group was the most increased, followed by *mmu_circ_0001335* (1.93‐fold, *P* < 0.05) and *mmu_circ_0001843* (1.90‐fold, *P* < 0.05), whereas *mmu_circ_0000607* (1.97‐fold, *P* < 0.05) was the most down‐regulated one, and the next were *mmu_circ_0001816* (1.85‐fold, *P* < 0.01) and *mmu_circ_0001815* (1.54‐fold, *P* < 0.05) in turn (Fig. 2). It was indicated that microarray results met the conditions for further analysis.

### Functional prediction

**Fig. 3:**
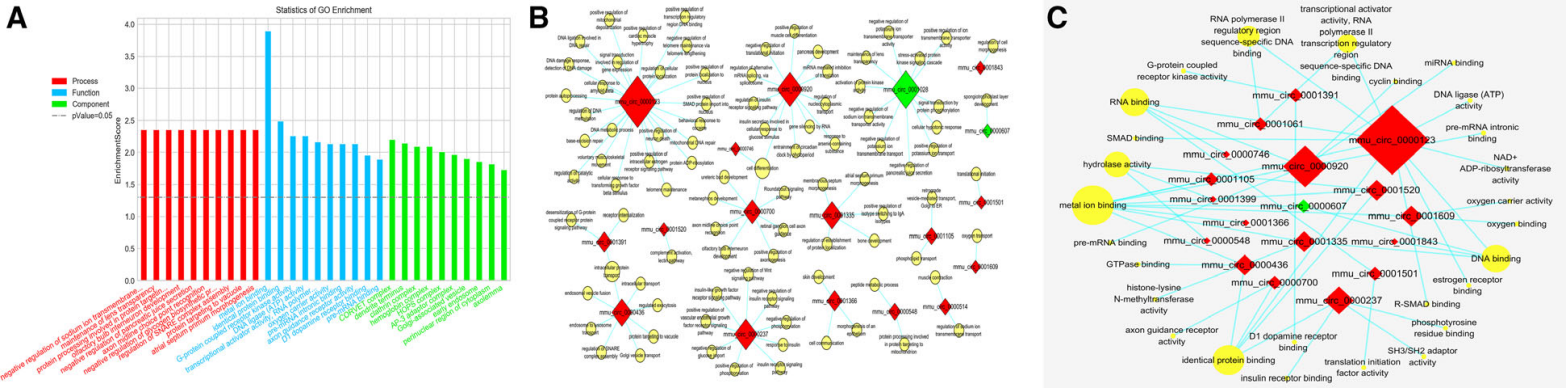
GO analysis for the host genes of all DECs. (A) GO annotation. Host genes according to the values in the enrichment score were annotated in the categories of BP, CC and MF, and the *x* and *y* axes represented the top 10 significantly enriched GO terms and degree of enrichment (gene ratio). (B, C) DECs BP (B) or MF (C) analysis. Red and green diamonds represented the up‐ and down‐regulated DECs. Yellow ellipse nodes indicated the predicted GO terms. The edges meant the regulatory relationships between the DECs and GO terms. The larger the yellow dot, the more DECs annotated in this term, and the bigger the red or green diamond, the more GO terms were involved for this DEC.

Based on the GO enrichment analysis, with the threshold of *P* < 0.05, the numbers of GO terms classified in CC, MF and BP were 19, 28 and 93, respectively. Although according to the threshold of FDR ≤ 0.05, there were 58, 0 and 1 GO terms annotated in the categories of BP, CC and MF. The top 10 GO terms (in ascending order of *P* value) of each category were laid out in Fig. 3A, and the most significant GO terms were ‘CORVET complex’ (CC, GO:0033263), ‘metal ion binding’ (MF, GO:0046872) and ‘negative regulation of sodium ion transmembrane transporter activity’ (BP, GO:2000650), severally. The molecular mechanisms and BP in which these DECs were probably involved were visualized in Fig. 3B,C to help further explore the potential roles of these DECs in the pathogenesis of AIH, and GO terms such as ‘cell differentiation’ (BP, GO:0030154), ‘intracellular protein transport’ (BP, GO:0006886), ‘RNA binding’ (MF, GO:0008380), ‘DNA binding’ (MF, GO:0003677) and ‘identical protein binding’ (MF, GO:0042802) were also closely connected with the regulation of these DECs.

**Fig. 4:**
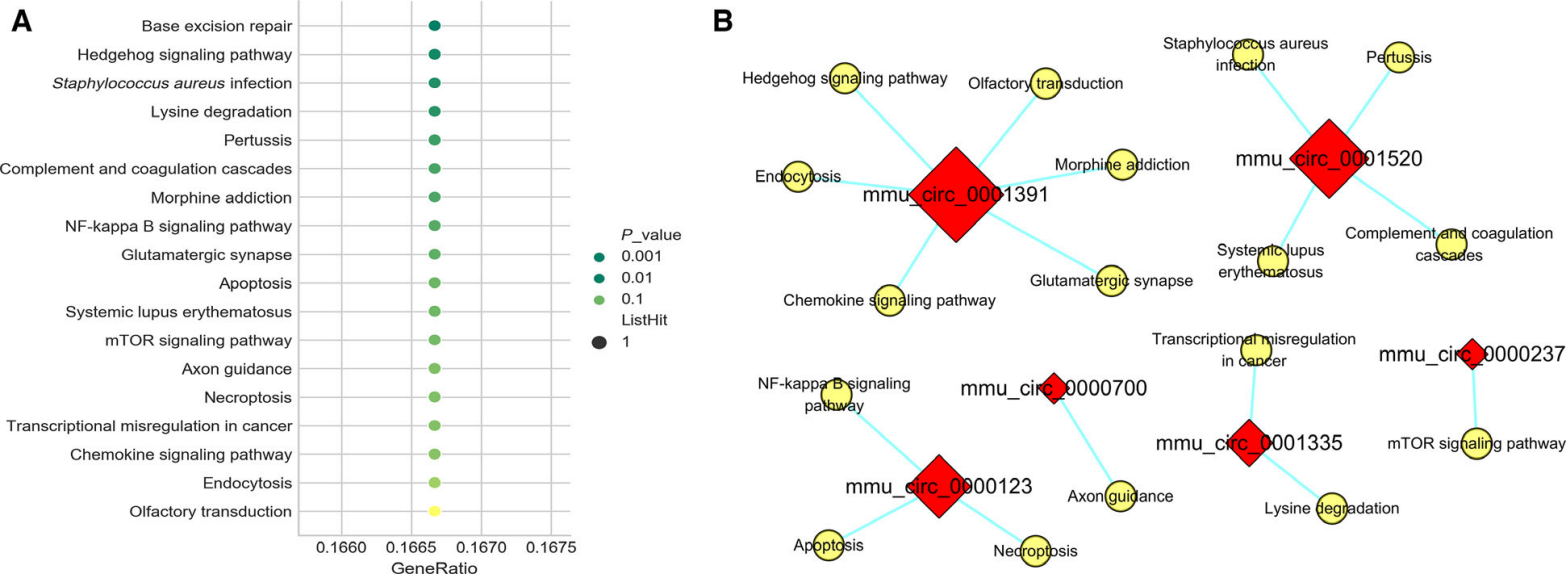
KEGG enrichment analysis for the host genes of all DECs. (A) Bubble diagrams of the top enriched KEGG pathways. The *x* and *y* axes represented pathways and degree of enrichment (gene ratio). The larger the dot, the more genes that enriched in this pathway. The greener the dot, the higher was the enrichment significance. (B) DECs pathways analysis. Only six circRNAs were annotated in the KEGG pathways. Red diamond represented the up‐regulated DECs. Yellow ellipse nodes indicated the predicted pathways. The larger the red diamond, the more pathways were enriched for this DEC.

For the results of KEGG analysis, six DECs were annotated in 19 signaling pathways, and the top 3 pathways ranked in ascending order by *P* value were ‘Base excision repair’ (path: mmu03410), ‘Hedgehog signaling pathway’ (path: mmu mmu04340) and ‘*Staphylococcus aureus* infection’ (path: mmu 05150) (Fig. 4A).

However, there was zero pathways annotated for these six DECs in line with the threshold of FDR ≤ 0.05. The ‘circRNA–pathway’ network was constructed in Fig. 4B, to visually reveal the possible process in which these DECs participated. *mmu_circ_0001391* and *mmu_circ_0001520* were annotated in six and four pathways, which was more than the other DECs. This indicated that they were likely to exert diverse biological functions in AIH.

### circRNA–miRNA analysis

**Fig. 5:**
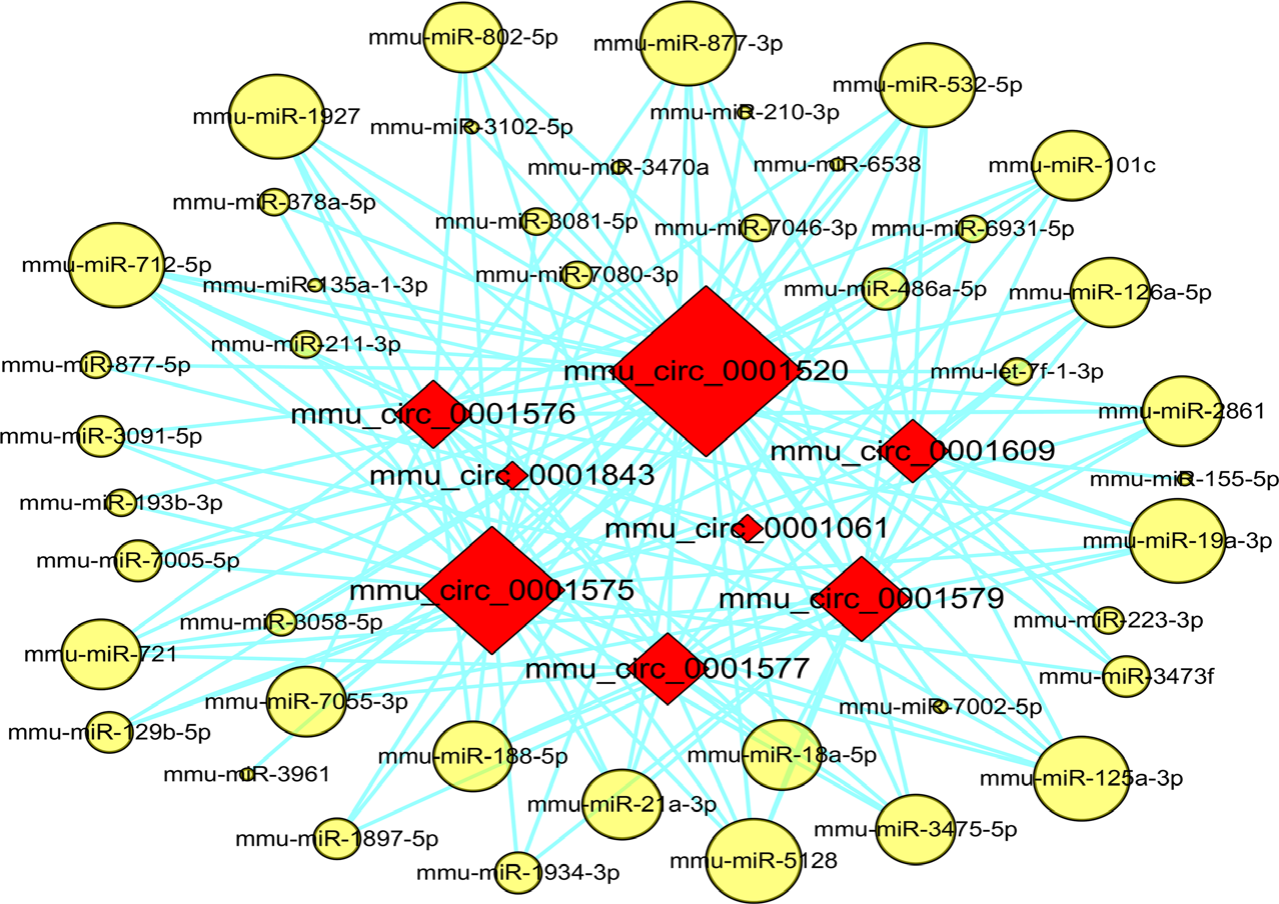
circRNA–miRNA pairing networks. Red diamonds represented the up‐regulated circRNAs. Yellow ellipse nodes indicated the target miRNAs. The edges meant the circRNA–miRNA pairing relationships. The larger the red diamond, the more miRNAs it interacted with, and the bigger the yellow dot, the more circRNAs it could pair.

**Fig. 6:**
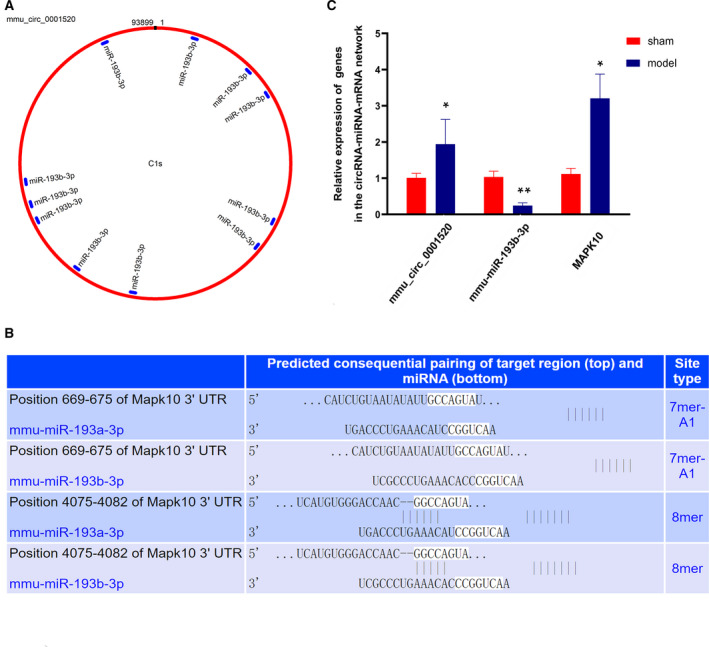
The predicted binding sites and validation of the *mmu_circ_0001520/mmu‐miR‐193b‐3p/MAPK10* network. (A) Predicted binding sites of *mmu‐miR‐193b‐3p* on the sequence of *mmu_circ_0001520*. The red circle represented the sequence of mmu_circ_0001520, and each blue short line meant the binding site for mmu‐miR‐193b‐3p. (B) Consequential pairing of *MAPK10* and *mmu‐miR‐193b‐3p*.The potential binding sites between *MAPK10* and *mmu‐miR‐193b‐3p* were shown according to the TargetScanMouse database (Release 7.2). (C) Relative expression of *mmu_circ_0001520*, *mmu‐miR‐193b‐3p* and *MAPK10*. The relative expression of *mmu_circ_0001520*, *mmu‐miR‐193b‐3p* and *MAPK10* in the model group were validated with qRT‐PCR (*n* = 4). *GAPDH* or *U6* was used as the internal control. The data were shown with the mean ± standard deviation (error bars) and statistically analyzed by Student's *t*‐test. *P < 0.05, **P < 0.01 compared with the sham group. *P* < 0.05 was considered statistically significant.

As appears in Fig. 5, the circRNA–miRNA network was constructed with 8 DECs and 43 target miRNAs according to the set threshold. The circRNA–miRNA interactions derived from the network contributed to further understanding the regulatory functions of these DECs. It is indicated that *mmu_circ_0001520* was indispensable in the pathogenetic process of AIH, because it was able to interact with the most miRNAs. In addition, we also predicted that the ‘*mmu_circ_0001520/mmu‐miR‐193b‐3p/MAPK10*’ network was probably involved in the development of this disease, and the forecasted consequential pairing relationship of *mmu_circ_0001520*/*mmu‐miR‐193b‐3p* and *mmu‐miR‐193b‐3p/MAPK10* was exhibited in Fig. 6A,B. The qRT‐PCR results revealed that (as shown in Fig. 6C), compared with the sham group, the expressions of *mmu_circ_0001520* (1.94‐fold, *P* < 0.05) and *MAPK10* (3.21‐fold, *P* < 0.05) were significantly increased in the model group, whereas the expression of mmu‐*miR‐193b‐3p* was decreased (4.33‐fold, *P* < 0.01).

## Discussion

At present, with more and more information available on the biological functions of circRNA, it has become a new hotspot in RNA research areas [30]. In the field of liver disease, studies have reported the associations between circRNA and nonalcoholic steatohepatitis, hepatic carcinoma, as well as the mechanism of liver regeneration [31, 32, 33]. However, the role of circRNA in AIH is still unknown because there has been little research on this topic.

Our study was the first stepping‐stone toward understanding the underlying part of circRNA taken in the nosogenesis of AIH. As mentioned earlier, a total of 27 DECs were found in the Con A‐induced AIH mouse model, and these DECs were clustered together and distinguishable visually from the circRNAs that did not meet the threshold (Fig. 1). Combined with the validation results of six chosen DECs with qRT‐PCR (Fig. 2), it was indicative that our microarray screening data were reliable for further functional predictions.

It was found that circRNAs originated from their host linear transcripts, and a host gene could be spliced into one or more circRNAs [34]. Therefore, it was assumed that the function of circRNA was related to the known effect of their host genes [35]. In this study, 21 of the 27 DECs originated from 21 host linear transcripts, whereas 6 DECs were intergenic (suggesting that they did not have the corresponding linear transcripts) (Table S1). To further investigate the regulatory role of these DECs in AIH, we performed GO analysis to annotate the biological imprints of host linear transcripts. Results of GO enrichment analysis manifested obviously that the regulatory function of DECs was most closely related with the response to ‘metal ion binding’ (MF, GO:0046872) and ‘negative regulation of sodium ion transmembrane transporter activity’ (BP, GO:2000650) (Fig. 3A), which had been acknowledged as the notable activities in the early stage of T cell‐mediated autoimmune diseases [36, 37, 38]. The GO terms ‘cell differentiation’ (GO:0030154) and ‘intracellular protein transport’ (GO:0006886) in the BP category (Fig. 3B), which were correlated to more than one DEC, were regarded as hub terms and were also thought to be associated with the occurrence of AIH [39, 40, 41]. Moreover, ‘RNA binding’ (MF, GO:0008380), ‘DNA binding’ (MF, GO:0003677) and ‘identical protein binding’ (MF, GO:0042802) were also the GO terms that were closely connected with the regulation of some DECs (Fig. 3C). It was stipulated that these screened DECS were able to exert important biological functions by acting as protein or miRNA inhibitors or sponges to regulate protein function or to be translated themselves, as well as to influence the DNA binding ability and lead to the increase or decrease of protein expression by forming post‐transcriptional regulators [42, 43].

Thereafter, the KEGG enrichment analysis was also performed to reveal the key signaling pathways that were possibly mediated by the linear transcripts. Coinciding well with the results of differentially expressed miRNAs in the same model (data not shown), KEGG pathway analysis displayed that these linear transcripts may be linked to the processes of endocytosis and apoptosis, including the ‘Endocytosis’ pathway (path: mmu04144), ‘mammalian target of rapamycin (mTOR) signaling pathway’ (path: mmu04150) and ‘Apoptosis’ (path: mmu04210). Endocytosis is a distinguished signal pathway concerning AIH via maintaining the immune balance. It is generally known that T cell receptors undergo cycles of endocytosis and recycling, and the balance between these processes ensures the expression and dynamics of T cell receptors needed for T cells to respond to various antigenic stimuli such that immune responses are efficient and do not cause autoimmunity [44]. The mTOR is a highly conserved serine/threonine protein kinase and can determine the outcome of adaptive immunity by endowing T cells with the ability to integrate a multitude of signals properly. Furthermore, it was evident that mTOR may affect diverse processes in T cells by coordinately regulating immune receptor signaling pathways, metabolic programs and migratory activity [45]. It has been reported that the mitochondria‐mediated apoptosis and autophagy dysfunction in Con A‐induced hepatocyte injury *in vivo* and *in vitro* were connected with the Akt/mTOR signaling pathway [46]. Apoptosis is considered as a crucial component of various processes, including development of the immune system, and inappropriate apoptosis (either too little or too much) is a vital factor in the generation of AIH. On the one hand, data have divulged that liver selectively recruits and induces the apoptosis of activated CD8^+^ T cells after an immune response, whereas insufficient apoptosis in activated lymphocytes contributes to the onset of AIH. The T cell trapping was induced by specific chemokines and adhesion molecules. On the other hand, hepatocyte apoptosis, induced by autoreactive T cells following specific pathways, is able to be observed in humans and murine models throughout the liver parenchyma, mainly in the area of interface hepatitis [47, 48]. Therefore, changes in these signaling pathways, which probably have some relationship with these screened DECs, were supposed to be helpful in preparation to trigger AIH. However, all of these results were basically based on the bioinformatics prediction; thus, a much deeper study should be conducted in the future.

Because more and more evidence has proved that circRNAs regulate the function of miRNAs acting as the ceRNAs [49, 50, 51], circRNA–miRNA coexpression networks were constructed to predict the relationships between DECs and the differentially expressed miRNAs in the same model [26]. Eight DECs and 43 target miRNAs were involved in the established circRNA–miRNA coexpression network (Fig. 5), and it was exhibited that *mmu_circ_0001520* was likely to be the key regulating gene participating in the progression of AIH, for the reason that it has the potential to interact with 41 of the 43 miRNAs. Hitherto, no circRNA in the constructed circRNA–miRNA coexpression network has been reported to be associated with AIH, but some of the coexpressed miRNAs have already been proved to be crucially functional in this disorder. It was delineated that mmu‐miR‐155 modulated the differentiation of helper T (Th) 17 cells and regulatory T cells by targeting to suppressors of cytokine signaling‐1, a negative regulator of the interleukin (IL)‐2 signaling cascade [52], whereas mmu‐miR‐223 negatively regulated the expression of IL‐1β and suppressed proinflammatory activation of Kupffer cells at the early stage of Con A‐induced liver injury [53]. Furthermore, in view of the immune imbalance of T lymphocyte subsets being the hallmark of AIH [54], mmu‐miR‐210 (another coexpressed miRNA) was also considered to be related to the development of AIH because of its ability to skew the CD4^+^ Th cell‐mediated immune balance. It was characterized by not only inducing the differentiation of Th17 and Th1 cells but also inhibiting the differentiation of Th2 cells, via suppressing the expression of signal transducers and activators of transcription 6 and LYN (a cytoplasmic membrane‐associated tyrosine kinase) [55]. Taken together, these results suggested that circRNAs can play an essential role in controlling gene expression on the occurrence of AIH by binding up to miRNAs.

From the established circRNA–miRNA and miRNA–mRNA interaction network in the same model [26], a latent circRNA–miRNA–mRNA network ‘*mmu_circ_0001520/mmu‐miR‐193b‐3p/MAPK10*’ was constructed, and it was suggested that this predicted network was likely to be associated with the occurrence and development of AIH based on the literature research. Although no functional annotations of *mmu_circ_0001520* have been presented, complement component 1, s subcomponent 1, the best transcript of this DEC, was annotated in the GO terms of ‘complement activation, lectin pathway’ (GO:0001867) in the BP category. Meanwhile, C1s, as the enzymes initiating the activation of the classical pathway of complement involved in innate and adaptive immunity, are serum glycoproteins primarily produced by the liver and closely associated with liver development, function and regeneration [56, 57, 58]. In our study, *mmu_circ_0001520* was supposed to be the sponge of *mmu‐miR‐193b‐3p*, because many miRNA response elements were located on the sequence of *mmu_circ_0001520* (Fig. 6A). *mmu‐miR‐193b‐3p* was regarded as a new therapeutic target for Con A‐induced liver injury and fibrosis in that the expression of *mmu‐miR‐193b‐3p* was down‐regulated in liver tissues after exposure to Con A, and lentivirus‐mediated overexpression of *mmu‐miR‐193b‐3p* alleviated the liver injury by decreasing alanine aminotransferase and aspartate aminotransferase levels [59]. *MAPK10* was one of the reported target genes for *mmu‐miR‐193b‐3p* [60], and the predicted pairing relationship of *mmu‐miR‐193b‐3p/MAPK10* was specified in Fig. 6B. The protein [mitogen‐activated protein kinase 10 (MAPK10)] encoded by this gene is a member of the MAPK family. MAPKs act as an integration point for multiple biochemical pathways and thus are involved in the pathophysiological processes of inflammation in the liver by regulating the cytokines network (including interferon‐γ, tumor necrosis factor‐α, IL‐1β) [61]. Inhibiting the activation of MAPKs signaling pathway in some ways was reputed to be a promising candidate for therapy of AIH [62, 63].Therefore, it was indicated that *mmu_circ_0001520*, possibly acting as the sponge for *mmu‐miR‐193b‐3p*, may play a special role in the progression of AIH via elevating the expression of MAPK10. Examining the exact relationship between *mmu_circ_0001520*, *mmu‐miR‐193b‐3p* and *MAPK10* will be the subject of our future investigations.

## Conclusions

This paper provided the comprehensive landscape of circRNA expression profile related to AIH with a Con A‐induced mouse model for the first time. Based on the GO enrichment analysis, the host linear transcripts for these 27 DECs may be connected with the metabolism of cellular ions and influence on the protein expression by interacting with RNA, DNA or proteins. KEGG enrichment analysis was also performed and suggested that these DECs were likely to contribute to the processes of endocytosis and apoptosis. What is more, we found these DECs have the potential to regulate the function of miRNAs as a sponge, and a novel interaction network ‘*mmu_circ_0001520/mmu‐miR‐193b‐3p/MAPK10*’ was also predicted and validated by qRT‐PCR, which probably took part in the development of AIH. It will be helpful to make good use of the roles of circRNAs as ‘miRNA sponge’ to prevent the harms caused by this overexpression or low expression of miRNAs in patients with AIH. There is no doubt that a deep study on the molecular mechanism of circRNA is needed to provide further insights into the comprehension of AIH and to discover novel therapeutic targets for this disorder with great promise.

## Conflict of interest

The authors declare no conflict of interest.

## Author contributions

YL analyzed and interpreted the data, and wrote the original draft. ZL, JH and HC acquired the data. TH and HH conceived and designed the project, and reviewed and edited the paper. All authors read and approved the manuscript.

## Supporting information


**Table S1.** Features of the 27 DECs.

## Data Availability

All of the raw data are available from the corresponding author upon reasonable request.
