# Supplementary material for: Circular RNAs associated with a mouse model of concanavalin A‐induced autoimmune hepatitis: preliminary screening and comprehensive functional analysis
Source: FEBS Open Bio. 2020 Oct 14;10(11):2350–62. doi: 10.1002/2211-5463.12981 (PMC7609805; doi:10.1002/2211-5463.12981)
Supplement: Supplementary file 1 — Table S1. Features of the 27 DECs. [file FEB4-10-2350-s001.pdf]

Supplementary Table 1. Features of the 27 DECs

circRNA ID	BestTranscript	Genesymbol	Annotation	Chromosome	FC	*P*-value	Regulation
mmu_circ_0000920	NM_009032	Rbm4	INTERNAL, UTR3	chr19	9.07	3.66E-02	up
mmu_circ_0001520	NM_001097617	C1s1	UTR5	chr6	3.95	1.22E-05	up
mmu_circ_0000700	NM_175549	Robo2	INTERNAL	chr16	3.68	2.10E-04	up
mmu_circ_0001335	NM_001081102	Nsd2	OVEXON, UTR5	chr5	3.41	3.72E-02	up
mmu_circ_0001843	NM_001114119	Qrich1	INTERNAL	chr9	3.37	6.46E-03	up
mmu_circ_0001501	NM_025829	Eif4e3	INTERNAL	chr6	3.36	3.86E-03	up
mmu_circ_0000514	NM_032008	Slmap	INTERNAL	chr14	2.79	3.41E-02	up
mmu_circ_0001061	NM_013720	Mga	AINTERNAL	chr2	2.66	2.62E-03	up
mmu_circ_0000548	NM_027436	Mipep	INTERNAL	chr14	2.64	5.04E-04	up
mmu_circ_0000237	NM_001177629	Grb10	INTERNAL, UTR3	chr11	2.64	8.61E-06	up
mmu_circ_0001609	NM_008219	Hbb-bh1	UTR5	chr7	2.58	1.23E-02	up
mmu_circ_0000436	NM_172120	Vps41	INTERNAL	chr13	2.54	1.50E-02	up
mmu_circ_0001366	NM_175473	Fras1	INTERNAL	chr5	2.51	1.60E-03	up
mmu_circ_0001577	None	None	INTERGENIC	chr7	2.35	9.04E-05	up
mmu_circ_0001391	NM_177078	Grk3	INTERNAL	chr5	2.33	2.03E-02	up
mmu_circ_0001575	NR_015351	Ipw	upstream_start	chr7	2.29	1.66E-03	up
mmu_circ_0000078	None	None	INTERGENIC	chr1	2.26	1.73E-02	up
mmu_circ_0000123	NM_007415	Parp1	INTERNAL	chr1	2.16	3.19E-03	up
mmu_circ_0001105	NM_015731	Atp9a	INTERNAL	chr2	2.15	1.66E-02	up
mmu_circ_0001576	None	None	INTERGENIC	chr7	2.11	7.58E-04	up
mmu_circ_0001579	None	None	INTERGENIC	chr7	2.04	2.86E-03	up
mmu_circ_0001399	NM_023625	Plbd2	INTERNAL	chr5	2.04	9.70E-03	up
mmu_circ_0000746	NM_001199044	Prrc2a	INTERNAL, intronic	chr17	2.00	7.39E-04	up
mmu_circ_0001816	None	None	INTERGENIC	chr9	-2.21	3.37E-03	down
mmu_circ_0000607	NM_001145888	Zfat	INTERNAL	chr15	-2.30	4.69E-02	down
mmu_circ_0001815	None	None	INTERGENIC	chr9	-2.40	9.42E-04	down
mmu_circ_0001028	NM_016866	Stk39	INTERNAL	chr2	-4.89	3.02E-02	down
